# Clinical application and technical details of cook zenith devices modification to treat urgent and elective complex aortic aneurysms

**DOI:** 10.1186/s42155-021-00233-7

**Published:** 2021-06-01

**Authors:** Jesse Manunga, Lia Jordano, Aleem K. Mirza, Xiaoyi Teng, Nedaa Skeik, Laura Eisenmenger

**Affiliations:** 1 Section of Vascular and Endovascular Surgery, Minneapolis Heart Institute at Abbott Northwestern Hospital, 920 E 28th Street, Ste 300, Minneapolis, MN 55407 USA; 2 Minneapolis Heart Institute foundation, University of Wisconsin at Madison, Minneapolis, MN USA; 3 Department of Radiology, Division of neuroradiology, University of Wisconsin at Madison, Madison, USA

**Keywords:** Physician modified stent graft, Complex aortic aneurysm, And endovascular repair

## Abstract

**Purpose:**

To describe technical details of modifying four different Cook Zenith devices to treat complex aortic aneurysms.

**Material:**

In the first three cases, the modification process involved complete stent graft deployment on a sterile back table. Fenestrations were created using an ophthalmologic cautery and reinforced with a radiopaque snare using a double-armed 4–0 Ethibond locking suture based on measurements obtained on centerline of flow. In each instance, a nitinol wire was withdrawn and redirected through and through the fabric and used as a constraining wire. In the fourth patient, modification involved partial stent graft deployment and creation of additional two fenestrations to accommodate renal arteries. The devices are resheathed and implanted in the standard fashion.

**Results:**

Four patients underwent exclusion of their aneurysms, including thoracoabdominal aneurysms (*n* = 2), a contained ruptured juxtarenal aneurysm (*n* = 1), and a ruptured failed previous endovascular repair (*n* = 1). Fifteen fenestrations were successfully bridged with Atrium iCAST stent grafts. Average graft modification time, operative time, contrast volume, radiation dose, estimated blood loss, and hospital length of stay were 89 min, 155.25 min, 58.8 mL, 2451 mGy, 175 mL, and 4.3 days, respectively. One patient required a secondary intervention to treat a type Ib endoleak. During an average follow-up of 25 months, aneurysm sacs progressively shrank without additional intervention.

**Conclusion:**

Physician-modified fenestrated/branched endografts are a safe alternative to custom made devices, especially in urgent cases and should be part of the armamentarium of any complex aortic program.

## Introduction

Ruptured abdominal aortic aneurysm is commonly a lethal condition; however, lower mortality has been observed in patients treated at high volume centers offering endovascular intervention. (Karthikesalingam et al., 2014) Unfortunately, a number of patients have aneurysms not amenable to approved infrarenal devices, and exclusion requires incorporation of visceral arteries. Compared to open repair, fenestrated/branched endografts (f/b-EVAR) confer lower mortality in patients with complex aortic aneurysm (cAAA). (Jones et al., 2019) However, manufacturing of custom made devices (CMD) takes up to 12 weeks, limiting their use in emergent situations. Off-the-shelf devices are approved in some countries, but clinical trials evaluating their safety are still underway in the United States (US). Furthermore, several studies have shown that only 58–88% of patients with cAAA meet inclusion criteria for off-the-shelf multibranched stent grafts. (Sweet et al., 2009; Park KHk Hiramota et al., 2010) Parallel grafts have been used to treat patients with cAAA. The technique is shown to be effective for patients treated with two snorkels. However, branched thrombosis is higher especially when three or more snorkels are used. For this reason, the European Society for Vascular and Endovascular Surgery does not recommend the use of more than two chimney grafts, limiting the use of this technique in patients requiring seal above the superior mesenteric artery (SMA). (Gupta et al., 2017)

These limitations can be overcome with the use of physician modified endovascular grafts (PMEGs); however, PMEGs are not widely adopted due to lack of training exposure and technical complexity. To aide understanding of PMEG procedural planning and execution, we describe our approach to the management of diverse cAAA pathology with PMEGs using four different Cook Zenith devices.

## Material and methods

Technical details of PMEG are illustrated in treating patients with different types of cAAA.

### Subject 1

An 85-year-old male was transferred from an outside institution (OSI) with an 8.5 cm contained rupture juxtarenal aneurysm (JRA). The aneurysm was successfully excluded with a three vessel PMEG using a Zenith Flex AAA Endovascular Graft Bifurcated Main body.

### Subject 2

A 70-year-old male was transferred from OSI with a symptomatic type B aortic dissection (TBAD) in a setting of a preexisting 6.7 cm extent IV thoracoabdominal aneurysm (TAAA). He initially underwent a thoracic stent graft placement using a Bolton Relay (Bolton Medical Inc. Sunrise, FL). At the one month follow-up, his aneurysm had grown to 7.6 cm. He underwent an urgent five vessels PMEG repair with a Zenith TX2 Dissection Endograft with Pro-Form and Zenith universal bifurcated body. A subsequent type Ic endoleak was treated with a Gore branched hypogastric device a month later.

### Subject 3

An 81-year-old male with a history of EVAR and multiple secondary interventions for endoleaks was transferred from an OSI with a ruptured right iliac artery aneurysm. He underwent extension of the repair into the external iliac but was also noted to have a type Ia endoleak. His aneurysm was excluded with a 3 vessels PMEG using the Zenith Alpha thoracic stent graft.

### Subject 4

A 68-year-old male was electively evaluated for a 6.8 cm extent V TAAA. The aneurysm was excluded with 4 vessels repair using a Cook Fenestrated Stent Graft (Zfen) modified by adding two fenestrations.

Additional details on the aneurysm type, cardiovascular risk factors, devices used, and the number of fenestrations are listed in Table 1.

**Table 1:** Aneurysm type, Risk factors and Modification specifications

Subject	Aneurysm type	Aneurysm size (cm)	Cardiovascular risk factors (CVRFs)	Device used	Number of fenestrations	Modification time (minutes)
1	JRAA	8.5	Hypertension Hyperlipidemia COPD Coronary artery disease Tobacco abuse ASA score: 4	Zenith Flex AAA Endovascular Graft Bifurcated Main Body	3 - SMA - RRA - LRA	98
2	TAAA	7.6	Hypertension Hyperlipidemia Obesity COPD Aortic Dissection ASA score: 4	Zenith TX2 Dissection Endograft with Pro-Form	5 - CA - SMA - RRAx2 - LRA	128
3	Failed previous EVAR	10.6	Hypertension Hyperlipidemia COPD CKD stage III Coronary artery disease Tobacco abuse ASA score: 4	Zenith Alpha Thoracic Stent Graft	3 - SMA - RRA - LRA	103
4	TAAA, extent V	6.8	Hypertension Hyperlipidemia COPD Coronary artery disease Tobacco abuse ASA score: 4	Cook Fenestrated Stent Graft (Zfen) with 2 existing fenestrations for CA and SMA	4 - CA - SMA - RRA - LRA	27

**JRAA**: Juxtarenal aneurysm; **TAAA**: Thoracoabdominal aortic aneurysm; **COPD**: chronic obstructive pulmonary disease; **CKD III**: chronic kidney disease, stage III; **ASA**: American Society of Anesthesiologists; **SMA**: superior mesenteric artery; **RRA**: right renal artery; **LRA**: left renal artery; **CA**: celiac axis

#### Technical details

**Fig. 1:**
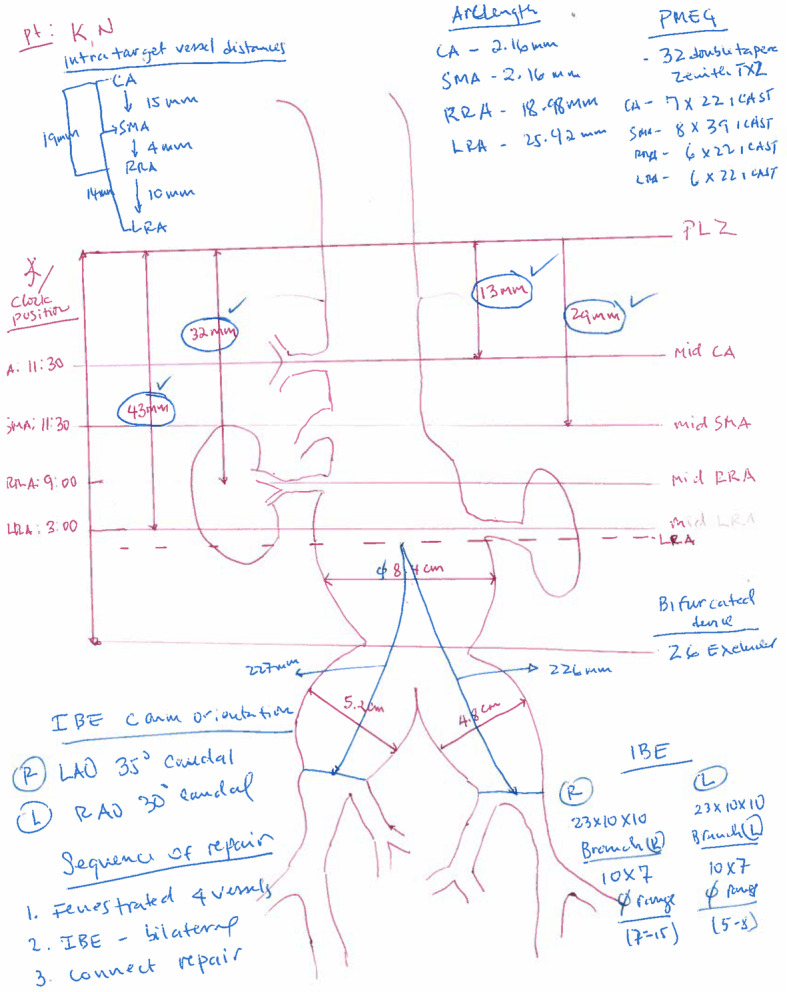
Preoperative planning sheet. See all measurements obtained using centerline of flow prior to taking the patient to the operating room. This includes the location of the proximal landing zone, inner vessel diameter, distance from the top of the graft to every target vessel, distance from the top of the graft to the bifurcation. Size of target vessels and bridging stent graft to be used. In the illustrated case, the patient had large bilateral common iliac aneurysms that were treated at the same time

Endograft choice, diameter, length, number, and location of fenestrations are based on review of the preoperative computed tomography angiography (CTA) analyzed in TeraRecon (Durahm, NC, USA) to obtain a centerline of flow and anatomic measurements. This is the first and most critical step of the PMEG procedure. For this reason, we routinely obtain at least two centerline of flow measurements to ascertain sizing accuracy. We start by determining the best proximal landing zone, defined as 2 cm of parallel aortic wall with no calcification or thrombus. The distance from the proximal landing zone to each target vessel is recorded with precision. Similarly, the distance between every target vessel (celiac artery (CA) to SMA), CA to each renal arteries) are recorded to facilitate measurements during stent graft modification (Fig. 1). In our experience, the CA and SMA are usually come off the aorta between the clock position of 11 and 1:30. The right renal tends to be located between 8:15 and 10 O’clock while the left renal usually comes off between 2 O’clock and 3:30. However, these vessels can come off at any clock position arguing for the need to customize every device to fit the patient’s anatomy. An inner vessel diameter measurement is needed to determine the position of the fenestrations. This diameter is based on the size of the aorta at the level of target vessels. For patients with thoracoabdominal aortic aneurysms, the size of stent graft in the visceral segment should be used as the inner vessel diameter since the aortic segment is aneurysmal. The clock position is recorded for every target vessel to be incorporated and is used to calculate arc length (2 *πr*(Θ/360) or the distance, in millimeters, from the middle (12 O’clock) of the graft to the exact location of the fenestration. This work must be done before starting graft modifications.

**Fig. 2:**
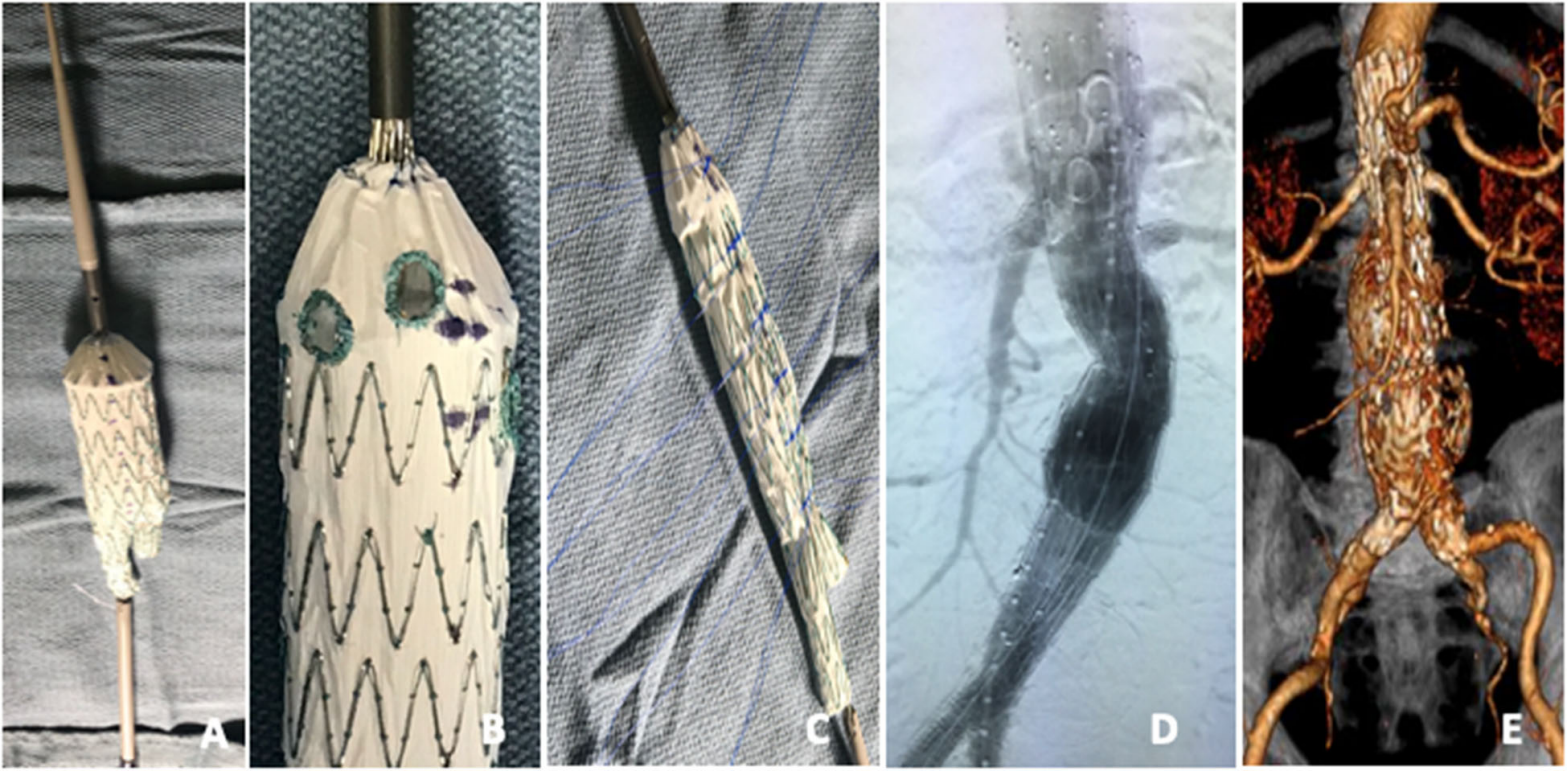
Steps involved in the modification of the Zenith Flex AAA bifurcated main body. The device is deployed on a sterile back table and one of the 3 nitinol wires is withdrawn from the inner cannula (**A**). Fenestrations are created using an ophthalmologic cautery and reinforced with a radiopaque snare using 4–0 Ethibond locking sutures (**B**). The nitinol wire is redirected through and through the fabric and the device is constrained every Z stent using the nitinol wire for support and two non-locking polypropylene loops prior to being resheathed (**C**). Completion angiography showing exclusion of the aneurysm and patency of all target vessels (**D**). 3D CTA obtained 24 months post-operatively showing continued patency of target vessels and aneurysm exclusion (**E**). **Rationale for device selection**: In a contained rupture JRAA amenable to 3 vessel PMEG, this two piece repair is ideal since, in case of frank rupture during implantation, the gate can be rapidly cannulated and contralateral limb placed, excluding the aneurysm prior to cannulating and bridging fenestrations. However, to avoid malalignment, we recommend bridging at least one fenestration (usually the SMA) prior to removing the diameter reducing tie. Provided sizing was accurate, one should still be able to cannulate and bridge renal artery fenestrations after the aneurysm is excluded

**Fig. 3:**
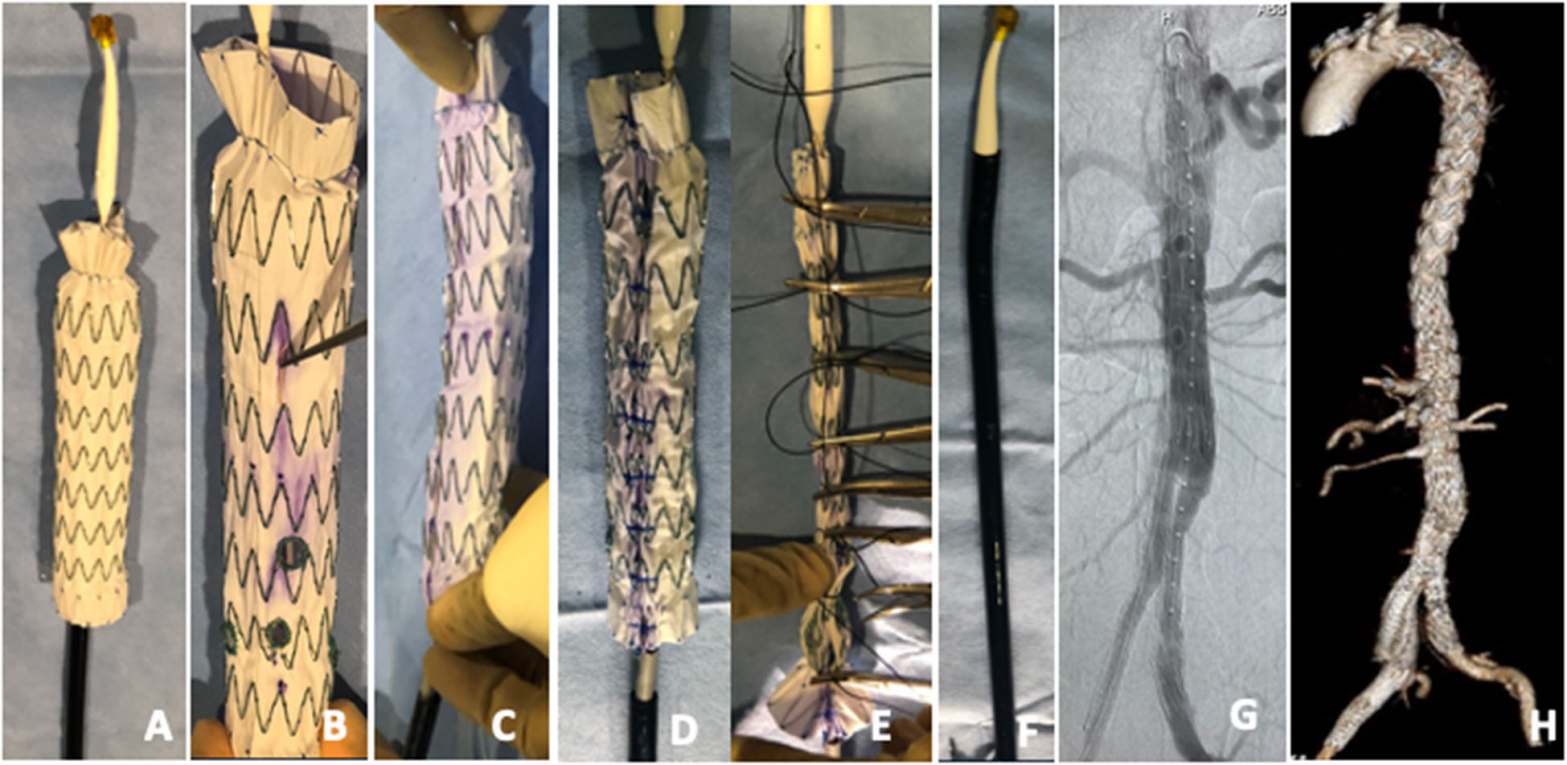
Steps of modification of the Zenith TX2 Dissection Endovascular Graft with Pro-Form. The device is deployed on a sterile back Table (**A**). Creation and reinforcement of fenestrations are as described in Fig. 5B. Note the presence of an anterior marker (not yet sutured in place) which aids with device orientation (**B**). **A** long spinal needle is used to redirect one of the 3 nitinol wires removed from the inner cannula through and through the endograft fabric (**C**). **T**he device is constrained posteriorly at every Z stent as described in Figure legend 2C (**D**). The graft is collapsed using silk ties and resheathed (**E&F**). Completion angiography and post-operative 3D CTA showing exclusion of the aneurysm and patency of all 6 target vessels (celiac, SMA, 2 right renal arteries, 1 left renal artery, and right internal iliac artery) (**G&H**). **Rationale for device selection**: The Zenith TX2 double tapered (32–24-158) was chosen for its size and length (32 mm diameter into a 30 mm diameter existing graft and 158 cm long) to allow seal into the existing TEVAR with a minimum of 3 stent overlap while providing adequate room for creation of five fenestrations. The distal tapered (24 mm diameter) allowed for the use of a Zenith fenestrated universal bifurcated device we had available in our inventory

**Fig. 4:**
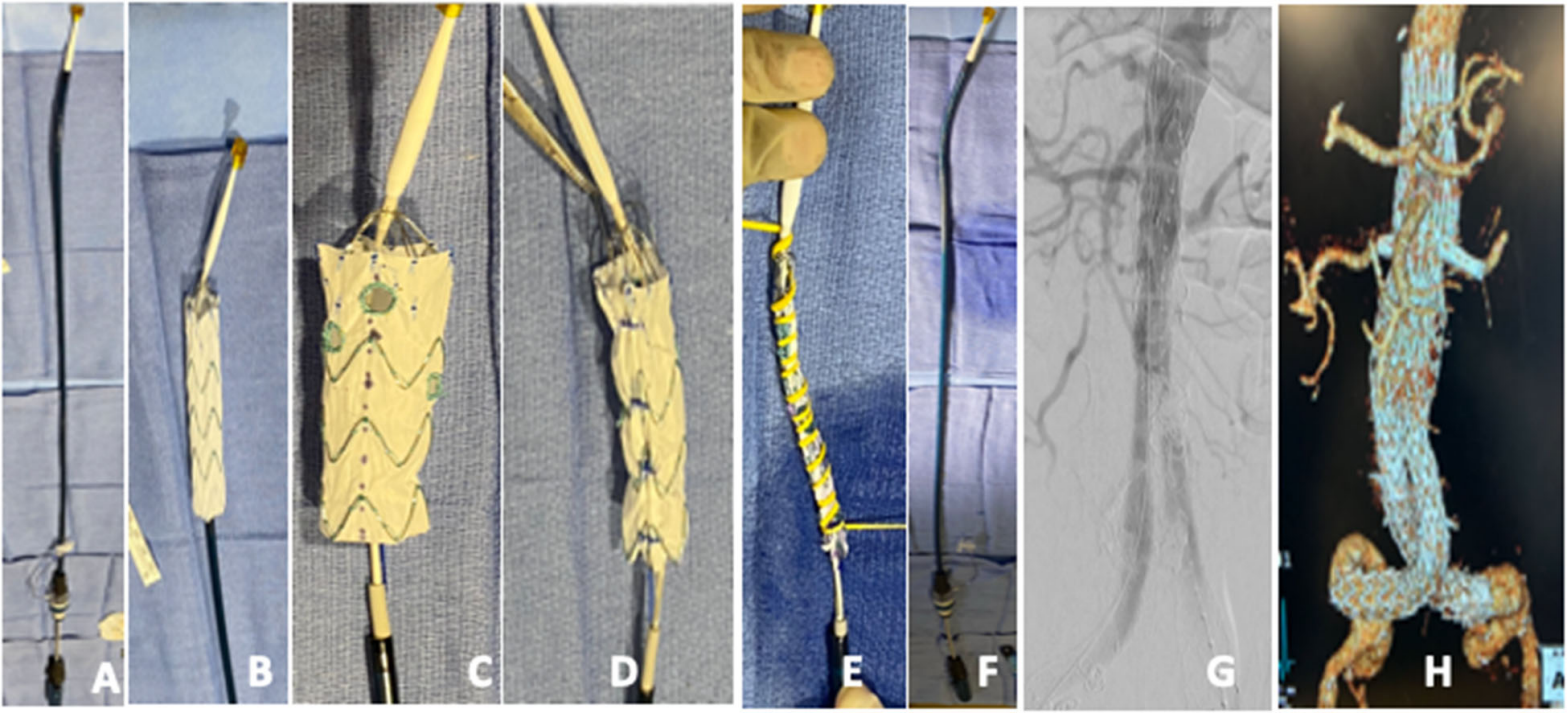
**A-H.** Steps of modification of the Zenith Alpha thoracic stent graft. The device is deployed on a sterile back Table (**A**). The bottom stent is cut with an ophthalmologic cautery to ensure adequate length to the flow divider of the failed stent graft. Fenestrations are created and reinforced as described in Fig. 1B (**B**). The device is posteriorly constrained at every Z stent (**D**), collapsed with a silastic loop (**E**), and resheathed (**F**). Completion angiography (**G**) and 3D post-operative CTA (**H**) show exclusion of the aneurysm, perfusion of target vessels, and no endoleak. **Rationale for device selection:** The existing stent graft was 24 mm in diameter, the visceral aorta measured 27 mm and the distance from the bottom of the celiac artery to the flow divider was 92 mm. Furthermore, iliac arteries were small and diseased. For this reason, the low profile Alpha thoracic stent graft ZTA-PT-30-26-108-W was the perfect fit for this 3 vessel repair after removal of the distal Z stent. Resheathing of this device requires removal of laser cut barbs, a process that is straightforward

**Fig. 5:**
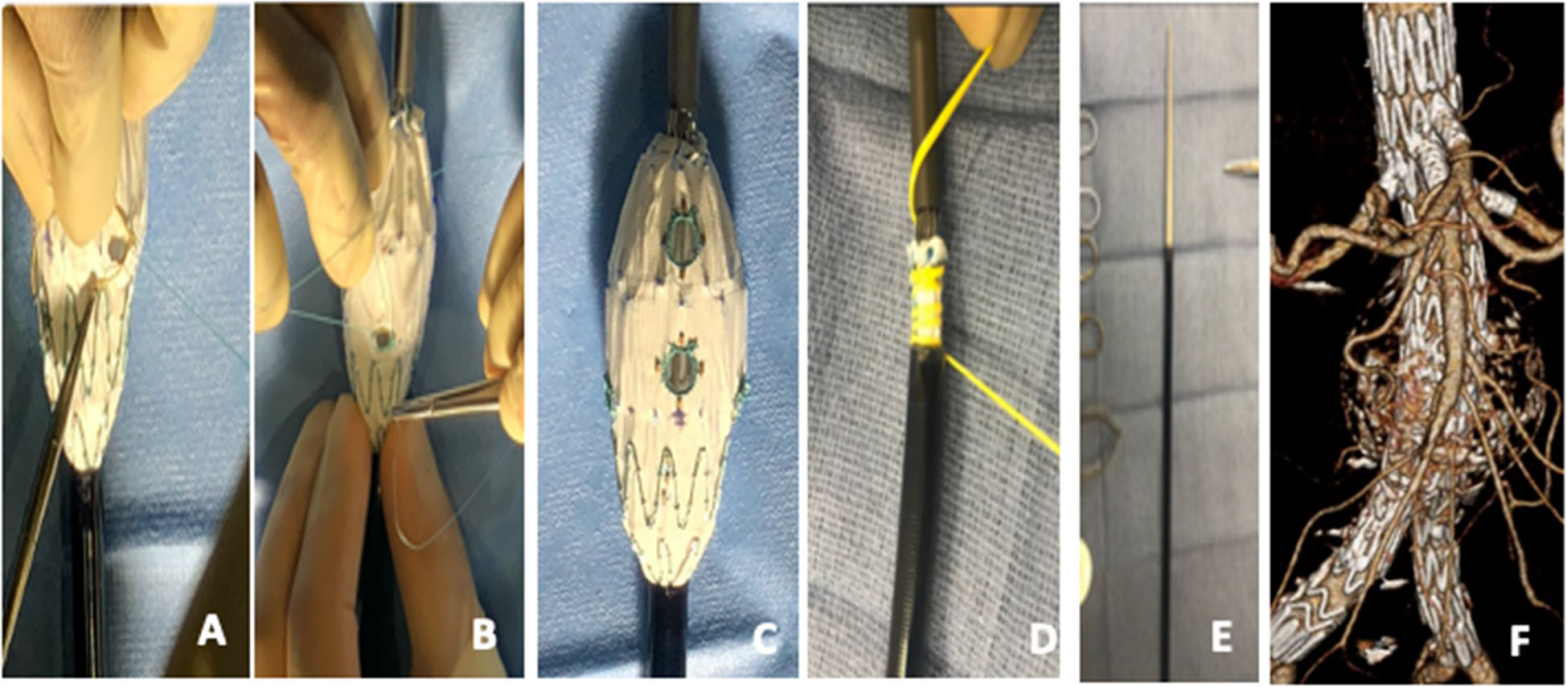
**A-F**. Steps involved in modification of the Zenith fenestrated stent graft (Zfen). The device is already constrained posteriorly and comes with 2 fenestrations created by the manufacturer to accommodate the celiac and superior mesenteric artery. Modification requires only partial deployment of the graft (**A**). Fenestrations are created and reinforced as described in Fig. 5C. (**A, B, and C**). The device is collapsed with silastic loops (**D**) and resheathed **(E**). Follow up post-operative 3D CTA confirm continued aneurysm exclusion and patency of target vessels. **Rationale for device selection**: Zfen is by far the easiest device to modify as it is already constrained and modification only requires addition of desired fenestrations and resheathing. This is our device of choice for all elective cases

Prior to patient induction, the chosen Zenith device is deployed on a sterile back table. Fenestrations are created using an ophthalmologic cautery and reinforced with a radiopaque snare and 4–0 double armed Ethibond suture in locking fashion. We prefer either 6 mm × 6 mm or 6 mm × 8 mm fenestrations, depending of the size of the target vessel and routinely use the Amplatz Goose Neck Snares (Medtronic, Minneapolis, MN) as our radiopaque markers to aid with fenestrations visualization under fluoroscopy and assist with target vessels cannulation. Once fenestrations are created, one of three nitinol wires is withdrawn at the base of the stent and redirected through and through the fabric using a long spinal needle. An anterior marker (radiopaque snare) is used to assist with determining correct device orientations in the patient prior to deployment. The device is constrained to 30% at every Z stent using the nitinol wire for support and two non-locking 3–0 polypropylene suture loops. The device is then collapsed with a combination of silastic loops and free ties and resheathed. Modification steps are illustrated in Figs. 2, 3, 4 and 5.

Further modification of the Zenith fenestrated stent graft involves partial unsheathing of the device just enough to create additional fenestrations using ophthalmological cautery. The process of fenestration reinforcement and device resheathing (Fig. 3) is similar to that described above.

#### Device implantation

Devices were implanted similar to previously published reports. (Manunga et al., 2019; Manunga & Titus, 2017; Manunga et al., 2018) The sole deviation from previous reports is the routine use of fusion technology to minimize contrast use.

## Results

Four patients underwent successful exclusion of their aneurysms with PMEGs using four different Zenith devices: three for symptomatic aneurysms and the fourth required an elective four vessel repair but was not a candidate for a US approved device. Device modification times and number of fenestrations created are detailed in Table 1. All fenestrations (*n* = 15) were successfully bridged with Atrium iCAST stent grafts. Average graft modification time, operative time, contrast volume, radiation dose, estimated blood loss (EBL), and hospital length of stay (HLOS) were 89 min, 155.25 min, 58.8 mL, 2451 mGy, 175 mL, and 4.3 days (2–5 days), respectively. Completion angiography in all four cases showed excellent exclusion of the aneurysm with no endoleak; however, one patient required a secondary intervention to treat a type Ib endoleak not originally detected. Over an average follow-up period of 25 months, aneurysm sacs continued to decrease with no additional secondary interventions.

## Discussion

A recent meta-analysis of patients with cAAA treated with PMEG show that the procedure has a technical success, 30-day mortality, and branched patency at 14.8 months ranging from 96.35 to 100%, 0% to 8%, and 96.3 to 100%, respectively. However, this study revealed several discrepancies between centers with regard to the type of stent used, device modification techniques, and the lack of reporting outcomes based on aneurysm types. For instance, for abdominal aortic pathologies, the stent graft used was reported in 273 (60%) cases, a strikingly low percentage. (Canonge et al., 2021) In a matched cohort of 82 patients with cAAA treated with either CMD or PMEG, Dossabhoy et al. reported no difference in perioperative complications, hospital length of stay, type I or III endoleak, or survival between the two devices. The only difference noted involved total fluoroscopy time, contrast volume used, and operative time. (Dossabhoy et al., 2018) Certainly, PMEG will continue to play an important role in the management of patients with cAAA for years to come. Unfortunately, the lack of a standardized protocol with regard of stent graft modification steps, sizing and type of bridging stent used by various centers performing these procedures make it impossible to perform a pooled or meta-analysis to help prove the long-term efficacy of this technique. Furthermore, the technique is not widely embraced owing to the lack of training and complexity of these operations.

While other devices have been successfully modified, the Cook Zenith remains our platform of choice for PMEG for several reasons. First, the devices are easily constrainable using one of the three nitinol wires located in the inner cannula of all Cook Zenith endografts, reducing the size and allowing device rotation in-situ to ensure fenestration alignment with target vessels. Second, the availability of straight and tapered devices of various sizes and lengths accommodates variable anatomy easily. Third, modification steps are similar for all devices.

In patients with tortuous vessels large enough to accommodate it, delivery of the PMEG through a previously placed Gore Dryseal sheath helps eliminate friction and ensures proper fenestration/target vessel alignment. This is also the case with failed EVAR being rescued with a PMEG. For this reason, we favor thoracic devices (Zenith TX2 TAA Endovascular Graft with Pro-Form or Alpha Thoracic Endovascular Endograft) for four vessels cases or for failed previous repair requiring 3 or more vessels incorporation due to the long delivery system. The Zenith Flex AAA bifurcated device is suitable for patients requiring one to three vessels repairs (Fig. 5) as the shorter delivery system makes it challenging to reach the celiac artery, especially in taller patients.

For patients requiring repair extension to the iliac arteries, we prefer the combination of a tapered Zenith thoracic device and Gore Excluder AAA Endoprosthesis or Iliac Branched Endoprosthesis (IBE) owing to their lack of suprarenal fixation struts that can crush bridging renal stents. We prefer to build our repair from the top down – the fenestrated cuff is placed first, followed by a bifurcated device. While acceptable, we often avoid a one-to-one size match between devices and allow for a minimum of two stents overlap between devices.

As illustrated by our carefully selected four cases, PMEG is indicated for a variety of patients with CAA, including those with infrarenal non amenable to currently approved devices, patients with juxtarenal, paravisceral, thoracoabdominal, certain patients with arch aneurysms and those with failed previous endovascular repair (failed EVAR). The technique is particularly useful in emergent or urgent situations, in patients who are poor candidates for open repair or those whose anatomy excludes them from being treated with currently approved CMD or off-the-shelf devices. However, not every patient is a candidate for PMEG; this includes patients with small and/or multiple renal arteries as well as those with excessive target vessels calcification. Furthermore, excessive aortic thrombus around target vessels may result in embolization to these vessels or lumbar arteries during device manipulation leading to renal impairment, bowel ischemia, or even paralysis. For this reason, careful patient selection is imperative.

Modification time is certainly an issue in emergent cases. The average device modification time for our four cases was 89 min, though considerably longer (109.7 min) for devices requiring posterior constraining and fenestration creation. For this reason, in situ fenestration is a reasonable approach in patients with frank rupture, though fenestrations are not reinforced. However, in our experience, back table modification operative metrics and mid-term outcomes are similar to patients treated with CMD in an elective setting.

From sizing to implantation, treating patients with cAAA with PMEG can be challenging. First, not an insignificant number of vascular specialists lack the training and expertise to expeditiously size a patient and obtain required measurements from software such as TeraRecon or 3mensio. Yet, we feel this critical skillset can be easily acquired by asking for a tutorial from software representatives. This step is key, and one should not attempt offering PMEG to patients without mastering it. Second, if not carefully planned, it is easy to find that the area where one of the four fenestrations needs to be created is not ideal due to the presence of a strut. For this reason, we mark, with a marking pen, the location of all fenestrations prior to starting burning fabric with an ophthalmologic cautery. There are instances when struts cannot be avoided. In this case, struts can be gently bent with a curved or straight hemostat prior to reinforcing the fenestration with a snare and Ethibond suture. Third, the Cook Alpha thoracic endograft has laser cut barbs in the proximal stent graft that prevent resheathing of the device. In this case, once can either cut these barbs with hemostat or can transition the modified device through a series of peel away dilators prior to resheathing it completely. The detailed steps of this technique have been previously described by Manunga. (Manunga, 2018) With careful planning and expert execution, PMEGs provide an important repair option in the treatment of cAAAs.

## Conclusion

While the long-term performance still unknown, PMEG is a safe alternative to CMD and plays an important role in the treatment of cAAAs requiring urgent or emergent repairs.

## Data Availability

N/A

## Abbreviations

f/b-EVAR: fenestrated/branched enografts
cAAA: complex aortic aneurysm CMD custom made devices
OSI: outside institution
JRA: Juxtarenal aneurysm
TAAA: thoracoabdominal aortic aneurysm
PMEG: Physician modified endovascular grafts
TBAD: type B aortic dissection
